# Preparation of the Endometrium for Frozen Embryo Transfer: A Systematic Review

**DOI:** 10.3389/fendo.2021.688237

**Published:** 2021-07-09

**Authors:** Sezcan Mumusoglu, Mehtap Polat, Irem Yarali Ozbek, Gurkan Bozdag, Evangelos G. Papanikolaou, Sandro C. Esteves, Peter Humaidan, Hakan Yarali

**Affiliations:** ^1^ Department of Obstetrics and Gynecology, Hacettepe University School of Medicine, Ankara, Turkey; ^2^ Anatolia IVF and Women Health Centre, Ankara, Turkey; ^3^ Nature IVF Unit, Thessaloniki, Greece; ^4^ Androfert, Andrology and Human Reproduction Clinic, Referral Center for Male Reproduction, Campinas, Brazil; ^5^ Department of Clinical Medicine, Aarhus University, Aarhus, Denmark; ^6^ The Fertility Clinic, Skive Regional Hospital Resenvej 25, Skive, Denmark

**Keywords:** frozen embryo transfer, hormone replacement treatment cycle, natural cycle, true natural cycle, modified natural cycle, individualized approach

## Abstract

Despite the worldwide increase in frozen embryo transfer, the search for the best protocol to prime endometrium continues. Well-designed trials comparing various frozen embryo transfer protocols in terms of live birth rates, maternal, obstetric and neonatal outcome are urgently required. Currently, low-quality evidence indicates that, natural cycle, either true natural cycle or modified natural cycle, is superior to hormone replacement treatment protocol. Regarding warmed blastocyst transfer and frozen embryo transfer timing, the evidence suggests the 6^th^ day of progesterone start, LH surge+6 day and hCG+7 day in hormone replacement treatment, true natural cycle and modified natural cycle protocols, respectively. Time corrections, due to inter-personal differences in the window of implantation or day of vitrification (day 5 or 6), should be explored further. Recently available evidence clearly indicates that, in hormone replacement treatment and natural cycles, there might be marked inter-personal variation in serum progesterone levels with an impact on reproductive outcomes, despite the use of the same dose and route of progesterone administration. The place of progesterone rescue protocols in patients with low serum progesterone levels one day prior to warmed blastocyst transfer in hormone replacement treatment and natural cycles is likely to be intensively explored in near future.

## Introduction

Over the last decade, efficient and safe vitrification techniques, alongside an increase in “freeze-all” cycles have contributed to a marked increase in frozen embryo transfer (FET) cycles globally (1). The number of FET cycles started to surpass fresh transfer in the United States and Australia and New Zealand in 2015 and 2016, respectively (1). Among all embryo transfers, the proportion of FET cycles was 77.0% as reported by the most recent update from the United States nationwide database (2). In Europe, a similar trend, however with a certain delay is seen; among all embryo transfers, the proportion of FET cycles increased from 28% in 2010 to 34% in 2016 (3, 4). The main contributor of the increasing trend for FET is an increase in freeze-all cycles; in 2016, of all oocyte retrievals, the rates of freeze-all were 26.5%, 19.2% and 8.5% in Australia and New Zealand, United States and Europe, respectively (1). The increasing trend to perform more pre-implantation genetic testing cycles for aneuploidy, especially in the United States (2), has also contributed to an increase in the total number of FET cycles (1).

Despite the increase in FET, the most optimal priming protocol of the endometrium is still a matter of debate (5). The available FET protocols are i) true natural cycle (t-NC) with/without luteal phase support (LPS) ii) modified NC (modified-NC) with/without LPS, iii) hormone replacement treatment (HRT) with or without gonadotropin releasing hormone (GnRH) analogue suppression, and iv) mild ovarian stimulation (mild-OS) using gonadotropins, clomiphene citrate (CC), or letrozole (**Table 1**).

**Table 1:** Endometrium preparation protocols for frozen embryo transfer (FET).

**Hormone replacement treatment (HRT)**
•HRT with GnRH-a suppression	
•HRT without GnRH-a suppression	
**True natural (t-NC) cycle**
•t-NC with luteal phase support
•t-NC without luteal phase support	
**Modified natural (modified-NC) cycle**
•Modified-NC with luteal phase support
•Modified-NC without luteal phase support
**Mild ovarian stimulation (Mild-OS)**
•Clomiphene Citrate (CC)/Aromatase Inhibitor (Letrozole)/Follicle Stimulating Hormone (FSH)

In the current systematic review, we aim to compare different FET protocols in terms of reproductive, obstetric and maternal outcomes. Following an overview of the available FET protocols, a special emphasis will be given to suggest an individualized approach if live birth rates (LBRs) are to be increased.

## Materials and Methods

PubMed and EMBASE databases were searched for studies that included the keywords (endometrial preparation, frozen embryo transfer, oocyte donation, egg donation, cryo-thawed, natural cycle, frozen embryo transfer, modified natural cycle embryo transfer, artificial frozen cycle, artificial frozen cycle with gonadotropin suppression) and MeSH terms (cryopreservation and pregnancy). The search was restricted to manuscripts published in peer review journals and abstracts in English language during 1991-2021. To review the topic of “Endometrium preparation for FET” advantages/disadvantages of different FET protocols and the potential impact of different FET protocols on reproductive outcomes are discussed based on the data, derived firstly from randomized controlled trials (RCT), meta-analyses, and secondly from large prospective cohort studies, whenever available.

## Endometrial Priming Protocols for Frozen Embryo Transfer

The two main endometrial priming protocols for FET are HRT and natural cycle (NC); less commonly, mild-OS is employed (6). In the HRT cycle, suppression of follicular growth, endometrial proliferation and subsequent secretory transformation is achieved by the timely administration of exogenous estradiol (E_2_) and progesterone (P). In NC, however, endogenous E_2_ and P secreted in a spontaneous cycle will prime the endometrium.

A FET cycle could be considered immediately after a failed fresh transfer cycle, as this results in a similar clinical pregnancy rate (CPR) to that postponed to a later time (7). Moreover, performing a FET cycle without delay will reduce the time to pregnancy and the psychological burden associated with waiting (8).

### Hormone Replacement Treatment (HRT)

#### Estrogen Administration

Treatment with oral E_2_ is started on the first, second or third day of the cycle to prime the endometrium and suppress spontaneous follicle growth. Estradiol is administered either at a fixed constant dose (6 mg daily) or in an incremental fashion; although many regimens have been used for the incremental increase of E_2_, the most commonly used is 2 mg/day during days 1-7, 4 mg/day during days 8-12, 6 mg/day during days 13 to embryo transfer (9). Although no RCT compared the fixed and step-up regimens, a large retrospective study performed in 8,254 oocyte donation cycles reported comparable LBRs (33.0 *vs* 32.5%, respectively) (9). In terms of estrogens, natural as well as synthetic E_2_ can be used, but again to our knowledge, no trial compared these two forms of E_2_ usage in HRT cycles. Different routes, including oral (micronized estradiol or estradiol valerate), vaginal (estradiol valerate) or transdermal (estradiol gel), can be used for the administration of E_2_ with comparable reproductive outcomes (5). Although vaginal E_2_ may be administered in a ring, tablet or cream forms, local vaginal irritation, discomfort, and unsatisfactory absorption especially when administered together with vaginal P are the reasons why the vaginal route of E_2_ administration is not the preferred route by the majority of IVF clinics (10). Thus, an international survey, conducted at 179 fertility units in a total of 56 countries, reported that, in 39,152 FET cycles, the oral E_2_ route was the most commonly used (84%), followed by transdermal (9%) and vaginal (3%) routes (6).

The conversion of doses, using different routes/types can be calculated as follows: 0.75 mg oral micronized estradiol = 1.25 g of transdermal estradiol gel = 1 mg oral/vaginal estradiol valerate (10). Importantly, a recent meta-analysis reported no difference between transdermal E_2_ and oral E_2_ as regards CPR (OR=0.86, 95% CI 0.59 - 1.25; n=504; 3 studies; low-quality evidence, I^2^ = 58%) and miscarriage rates (OR=0.55, 95% CI 0.27 - 1.09; n=414; 2 studies; low-quality evidence; I^2^ = 0%) (5).

The detrimental impact of elevated day 2/3 serum P_4_ levels on pregnancy rates in HRT-FET cycles has not been clearly elucidated. Although, data is limited, elevated basal levels of serum P_4_ levels (>1.5 ng/ml) does not seem to have negative impact on reproductive outcome (11). Usually, after 12 – 14 days of E_2_ administration, vaginal ultrasound examination is performed for endometrial thickness measurement and to confirm the absence of a leading follicle. This long E_2_ priming period may be unnecessary, as 5–7 days may suffice for adequate endometrial priming according to early studies (10, 12). Caution, however, is warranted, since a higher miscarriage rate has been reported using a shorter E_2_ priming period (<10 days) (13). Conversely, E_2_ administration may be prolonged for up to 28 days (14) or even 36 days (15), if necessary, without compromising reproductive outcomes and, thus, offering a greater flexibility of timing of FET (14). When the endometrial thickness >7 mm, P supplementation is commenced, and timing of FET is scheduled accordingly.

Suppression of the hypothalamus-pituitary axis by a GnRH-analogue can be performed prior to HRT. Although GnRH-agonists are commonly used for this purpose, to our knowledge, there is only one RCT comparing a 7-day regimen with daily GnRH-antagonist administration (n=287) to a single dose of long-acting GnRH-agonist (n=276) in an oocyte donation model, reporting similar ongoing pregnancy rates (OPRs) (46.7% *vs* 39.9%, respectively; adjusted OR= 1.42, 95%CI 0.97 - 2.09) (16).

Although HRT with suppression is highly efficient to avoid ovulation, HRT without suppression is more patient friendly. However, premature ovulation leading to cycle cancellation, encountered in 1.9% to 7.4% of the cycles, could be the main drawback of an HRT protocol without suppression (17, 18). In a recent Cochrane meta-analysis (5), only one RCT comparing LBR in HRT cycles with or without GnRH-agonist suppression was identified. In this trial, GnRH suppression improved LBR per initiated cycle (20.0% *vs* 8.5%; OR=2.62, 95% CI 1.19 - 5.78; n=234; one study; low-quality evidence) (19). However, the same Cochrane meta-analysis reported comparable CPR (OR=1.08, 95% CI 0.82 - 1.43; n=1289; eight studies; low-quality evidence, I^2^ = 20%), miscarriage rates (OR=0.85, 95% CI 0.36 - 2.00; n=828; four studies; low-quality evidence; I^2^ = 0%), cycle cancellation rates (OR=0.49, 95% CI 0.21 - 1.17; n=530; two studies; low-quality evidence; I^2^ = 0%) and endometrial thickness (MD -0.08, 95% CI -0.33; 0.16; n=697; four studies; low-quality evidence; I^2^ = 4%) with or without GnRH-suppression (5).

To conclude, in HRT cycle, E_2_ priming with oral or transdermal routes has similar efficacy. The optimal duration for E_2_ priming is between 10 to 36 days, which offers a greater flexibility of timing of FET without compromising reproductive outcomes. Although pituitary suppression with GnRH-agonist decreases the cycle cancelation rate, HRT without suppression is more patient friendly and is associated with similar CPRs when compared with those attained with GnRH-agonist suppression.

#### Progesterone Administration

Optimal exposure of P, in terms of timing and concentration, is crucial for the establishment and maintenance of an ongoing pregnancy. In this context, the route, dose and starting date of P should be taken into consideration. The available routes for P administration in an HRT cycle are vaginal, intramuscular (im), subcutaneous (sc), oral and rectal. The vaginal administration, distinct form the other routes, has a first-pass uterine effect (20–22), and until now, there is paucity of data on the use of oral (dydrogesterone) (23) sc (24) and rectal (25) routes.

Different forms of vaginal P can be used, including bio-adhesive gels, micronized tablets, capsules or suppositories. Of note, the typical doses used for vaginal P administration in an HRT cycle, are those extrapolated from fresh embryo transfer cycles; i) bio-adhesive P gel (1x90mg), ii) micronized P tablet (3x100 mg), iii) P capsule (3x200 mg), iv) suppository (2x400 mg). There is paucity of comparative data on different doses of vaginal P and the subsequent reproductive outcomes.

In a retrospective study, progesterone bio-adhesive gel once daily (90 mg/day; n=161) was compared with twice daily administration (90 mg/12 hours; n=185) in the HRT cycle, and both the implantation (7.6% *vs* 20.2%, p=0.0001) and delivery rates (8.7% *vs* 20.5%, p=0.002) were significantly higher with the twice-daily regimen (26). Moreover, in a more recent retrospective study of 2,010 HRT-FET cycles, the use of 1200 mg P capsules was associated with a significantly higher CPR when compared with a daily dose of 900 mg (27). Definitely, more studies are warranted to delineate the optimum dosing of various vaginal forms of P. More importantly, in the personalized medicine era, as will be discussed in the “*Individualized FET Approach*” section, monitoring the luteal serum P_4_ level is crucial to increase reproductive outcomes.

Since there is no corpus luteum in an HRT cycle, all the available P derives from exogenous administration. Vaginal P is more commonly used in Europe whereas im P has been preferred in the US. The debate whether vaginal or im P is superior, in terms of the reproductive outcome, is still ongoing. Thus, some retrospective studies reported better reproductive outcomes using the im route (28, 29), whereas others reported similar outcomes (30, 31). In total there are three RCTs comparing the im and vaginal routes in HRT-FET cycles, and similar clinical pregnancy rates were reported in two RCTs (32, 33). More recently, a RCT using vitrified blastocyst transfer in HRT cycles compared the OPRs in three arms consisting of 200 mg vaginal tablet P twice daily, 50 mg daily im P, only, and 200 mg vaginal P twice daily supplemented with 50 mg im P every third day (34). In interim analysis, a significantly lower OPR was reported in the vaginal P-only group: 31% compared with the other two groups (50% and 47%), which was mainly caused by a significantly higher biochemical loss and miscarriage rate rather than a lower positive human chorionic gonadotropin (hCG) rate in the vaginal progesterone-only group. Hence, the vaginal P-only arm was prematurely terminated (34). Patient recruitment continued for the remaining two arms to result in a total of 1,125 patients (1,060 FET cycles) for the whole study. Very recently, the results of the final analysis were reported. The LBR was significantly lower in the vaginal P-only arm (27%) when compared with im P (44%) or vaginal P supplemented with im P every third day (46%); however, no significant difference was noted between im P or vaginal P supplemented with im P every third day (35). One of the limitations of that study is heterogeneity as regards timing of FET in the three arms; vitrified blastocysts were warmed and transferred on the 5^th^ day of P in the vaginal P administration (with or without im P), whereas in subjects assigned to the im P only group, vitrified blastocysts were warmed and transferred on the 6^th^ day of P administration. Such difference in scheduling may introduce bias as P exposure duration until the embryo was transferred differed among groups. Moreover, serum P_4_ levels were not available. In our recent retrospective cohort study of 475 consecutive, day-5/6 vitrified–warmed HRT cycles, we were not able to reproduce the findings of Devine et al. study (36). Thus, intramuscular supplementation with 50 mg P every third day, did not enhance the OPR compared to vaginal progesterone-only. Moreover, the route (vaginal *vs* im) was not an independent predictor of OPR when tested in a multivariate logistic regression analysis. However, unlike Devine’s study, progesterone vaginal gel (twice daily), but not a vaginal tablet, was used in our study.

A recent retrospective study by Vuong et al., in HRT cycles, compared reproductive outcomes between vaginal micronized P 400 mg twice daily plus oral dydrogesterone 10 mg twice daily (n=732) versus vaginal micronized P 400 mg twice daily alone (n=632) for LPS (37). Significantly higher LBR (46.3% *vs*. 41.3%, respectively, RR=1.30, 95% CI 1.01–1.68, p=0.042), and lower miscarriage rate (3.4% *vs*. 6.6%; respectively, RR=0.51, 95% CI 0.32–0.83; p=0.009) have been reported with vaginal micronized plus oral dydrogesterone when compared with vaginal micronized P only (37).

The optimal length of luteal support in HRT cycles has never been explored in a RCT, but from a physiological point of view, P should be continued until the luteo-placental shift occurs which is still a matter of debate (38–40), and generally the common practice is to continue until 10^th^-12^th^ weeks of gestation (6).

In conclusion, there is paucity of data on the impact of different routes (vaginal, im, sc, oral or rectal routes) of P administration on reproductive outcome in HRT cycles. Vaginal administration is the most commonly used route. Future studies are warranted to delineate the optimum dosing of various vaginal forms of P. Since there is no corpus luteum in HRT cycles, P should be continued until the 10^th^ 12^th^ weeks of gestation.

#### Day of Starting Progesterone Administration

The interaction between embryo and a receptive endometrium is a complex molecular process essential for successful implantation (41). It is generally considered that once P_4_ levels reach a critical threshold, they set into motion a well-timed and orderly secretory transformation of the endometrium leading to receptivity (42).

There is paucity of data on the impact of the length of the P exposure on the reproductive outcome. To our knowledge, there are four RCTs, three published in peer review journals (43–45) and one abstract (46). Of those 4 RCTs, embryo transfer was carried out at the cleavage stage in two trials (43, 44), whereas the remaining two had embryo transfer at the blastocyst stage (46). The earliest RCT, conducted in an oocyte donation model and transferring day-3 embryos, compared P start on the 2^nd^ (egg retrieval+1 day; Group C; n=91), 3^rd^ (egg retrieval day, Group B; n= 94) or 4^th^ (egg retrieval-1 day; Group A; n=97) day of P administration in the recipients (43). Ongoing pregnancy rates per embryo transfer were similar in all the groups except for a higher biochemical pregnancy rate in group A (12.9%) when comparing with groups B (6.6%) and C (2.3%) (43). In another RCT, when cleavage stage day 3 embryos were warmed and cultured overnight to day 4, and transferred on the 5^th^ (n=150) or 3^rd^ (n=150) day of P administration, similar CPR`s were noted [37/137 (27.0%) *vs*. 26/138 (18.8%) respectively, OR=1.6, 95% CI 0.9–2.82, p= 0.11] (44). However, the early pregnancy loss rate was significantly higher following embryo transfer on the 3^rd^ day of the P administration group [32/58 (55.2%) *vs* 21/58 (36.2%); OR=0.46, 95% CI 0.22–0.97, p= 0.04)] (44).

For the blastocyst stage transfer, as mentioned, there are two RCTs; one was presented as an abstract (46) and one was published in a peer review journal (45). In the study by Ding et al., frozen/thawed blastocysts were transferred on the 6^th^ (n=23) or 7^th^ day (n=26) of P administration and a higher CPR, OPR and implantation rate was reported in the day 6 group (60.9% *vs* 53.8%, 56.5% *vs* 50.0% and 40.7% *vs* 30.0%, respectively), but the differences did not reach statistical significance (p>0.05) (46). Moreover, a recent RCT compared the outcomes of blastocyst transfer on the 5^th^ (n=151) or 7^th^ (n=152) day of P administration and in that study LBRs tended to be in favor of the 5^th^ day of P administration, although not reaching statistical significance (31.1% *vs* 25.7%, OR=0.76, 95% CI 0.46 – 1.26) (45). The same group, in a recent retrospective cohort study, compared the reproductive outcome of day-5 and day-6 vitrified blastocysts transferred on the 6^th^ (n=346) or 7^th^ (n=273) day of P start (47). Although the pooled day-5 and day-6 blastocysts had comparable LBRs when transferred on the 6^th^ or 7^th^ P start day (36.6% for both groups), of interest, a subgroup analysis revealed that day-6 blastocysts had an insignificantly higher LBRs when transferred on the 7^th^ day of P (35.5% *vs* 21.5%, p=0.06); the higher LBR was mainly caused by a lower miscarriage rate when transferred on the 7^th^ day of P (21.4% *vs* 50.0%, p=0.02). Although the retrospective study design and subgroup analysis could be considered a limitation of the study the finding of an increase in LBR by delaying transfer of day-6 blastocysts for one day (7^th^ day of P start) is contrasted by common practice in which day-6 blastocysts are transferred on the same day as day 5 blastocysts (6^th^ day of P start). A future RCT exploring this issue is clearly warranted to draw firm conclusions.

**Figure 1:**
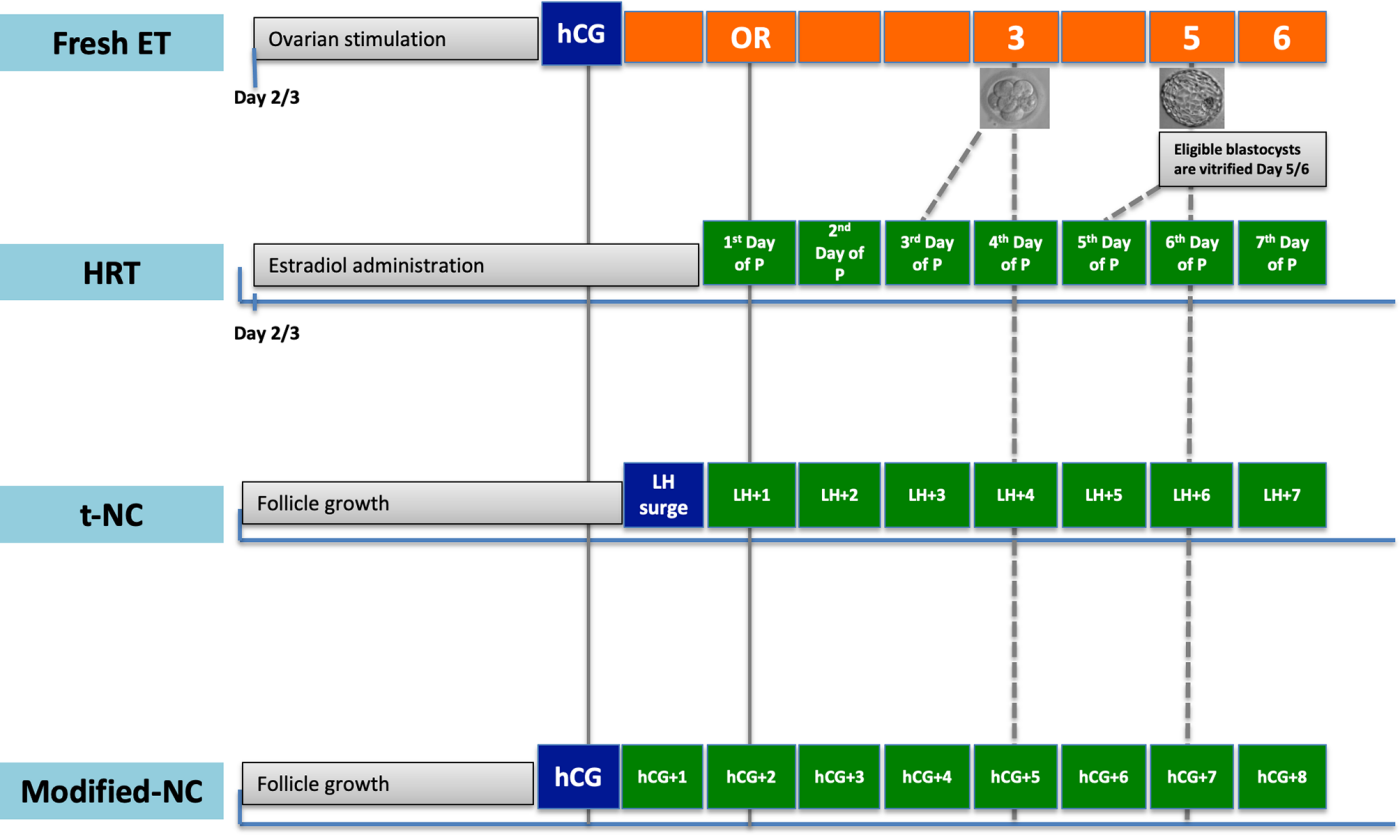
Timing of warmed embryo transfer in hormone replacement treatment (HRT), true natural cycle (t-NC) and modified natural cycle (modified-NC) protocols to prime endometrium. hCG, human chorionic gonadotropin; ET, embryo transfer; P, progesterone; OR, oocyte retrieval; LH, luteinizing hormone. The dash-lines denote timing of warmed embryo transfer in different FET protocols.

In conclusion, limited evidence suggests that, for the optimal length of P exposure before FET, Day-3 embryos should be transferred on the 3^rd^ or 4^th^ day of P administration and Day-5/6 blastocysts on the 5^th^ or 6^th^ day of P administration (**Figure 1**). A higher LBR for Day 6 vitrified blastocysts when transferred on the 7^th^ day of P administration, as reported by a single retrospective study, should be warranted by further RCTs.

### Natural Cycle (NC)

Both t-NC and modified-NC is most optimally performed in patients with regular menstrual cycles. In t-NC, to schedule FET, the timing of spontaneous ovulation needs to be precisely pinpointed, necessitating frequent endocrine and transvaginal ultrasonographic monitoring. Hence, NC is less flexible when compared with HRT and modified-NC. In modified-NC, triggering is performed when the leading follicle is between 16-20 mm in diameter and scheduling is performed accordingly; modified-NC requires less endocrine and ultrasonographic monitoring when compared with t-NC and, thus, is considered more patient-friendly.

#### True-NC (t-NC)

For t-NC, transvaginal ultrasonography is performed on day 2 or 3 of menses to rule out any cyst or corpus luteum prevailing from the previous cycle. Cycle cancellation is usually undertaken in cycles with serum P_4_ >1.5 ng/ml on day 2 or 3 of menses, even though this practice has been extrapolated from fresh embryo transfer cycles (48, 49). To our knowledge, there is no study evaluating the impact of day 2/3 serum P_4_ on success rates in t-NC, modified-NC, and mild-OS cycles. Transvaginal ultrasonographic monitoring is usually started on day 8-10 and endocrine monitoring is performed, using serum E_2_, LH and P_4_ measurements when the leading follicle attains a mean diameter of approximately 15 mm in diameter. Following frequent endocrine and ultrasonographic monitoring, on alternate days or daily, the day of ovulation is precisely documented to schedule the timing of FET.

The place for endocrine monitoring in t-NC is controversial. Thus, a retrospective study of 610 patients undergoing t-NC FET reported a 28.4% incidence of serum P_4_ elevation (>5 nmol/l) before the LH surge (50). No significant difference was noted in OPR (32.5% *vs* 31.7%) of those patients with or without P_4_ elevation on the day of LH surge (50). However, if P_4_ elevation lasted for 2 days, there was a significant reduction in the CPR (39.4% *vs* 20.7%, p=0.04). A subgroup analysis of that study suggested that, not the level, but the duration of P_4_ exposure before the LH surge was associated with the lower pregnancy rates (50).

#### Modified-NC

For modified-NC, the initial monitoring is the same as in t-NC; however, ovulation is triggered with hCG once the leading follicle reaches a mean diameter of 16-20 mm. In modified-NC, hCG, not only induces ovulation, but also results in increased serum P_4_ production during the early and mid-luteal phase, thus, the hCG trigger works as an ovulation trigger as well as an early LPS. To our knowledge, there is no study comparing different doses of hCG for triggering in modified-NC; in theory, the lowest effective dose to induce ovulation will result in a lower early serum P_4_, which should reduce the risk of endometrial advancement, known to have a negative impact on endometrial receptivity. The place for endocrine monitoring in modified-NC is controversial (51, 52). Whether monitoring of serum P_4_ and LH levels in modified-NC FET cycles has added clinical value needs to be explored.

#### How to Pinpoint the Day of Ovulation in t-NC?

Pinpointing the day of ovulation is of crucial importance in t-NC and will rely on documenting the LH surge and/or ultrasonographic signs of ovulation. The LH surge should be tested in serum rather than urine since urinary LH testing, with detection limits of 20-40 IU/L, is associated with high false-negative results (53). Moreover, with urinary testing when compared with serum testing, one-day delay should be taken into account for timing of FET due to urinary clearance of LH (54–56).

In the NC, the E_2_ synthesis increases progressively from the dominant follicle and triggers the LH surge. Prior to the LH-surge, a small increase in P_4_ level is encountered, which reflects the increasing LH pulse amplitude and frequency leading up to the surge (57). Different cut-off points have been described to define the LH surge; even different criteria may be employed in the same clinic (58). Most commonly, a rise of at least 80% above the latest serum LH level with a continued rise, thereafter, is defined as a surge of LH (54, 59). A drop in serum E_2_ follows the initiation of LH surge due to luteinization of granulosa cells and synthesis of P_4_ by rising LH levels. Another suggested definition is the first attainment of LH ≥17 IU/L during the follicular phase with a ≥30% drop in E_2_ levels, the following day (58). The highest serum LH level obtained (usually a day after LH ≥17 IU/L) has been considered by others to be the day of LH surge (58). Moreover, a serum LH >10 IU/L has also been described to define the LH surge (51). Clearly, this heterogeneity in definition may result in differences in timing of FET which may have impact on reproductive outcomes (58). Irrespective of the definition, a concomitant rise in serum P_4_ (>1.5 ng/ml) the day after the LH surge, should be seen to confirm ovulation.

For timing of FET, some clinics, in addition to documentation of the LH surge, also use ultrasonographic signs of ovulation. This may be of some relevance since, although ovulation usually occurs 24 h after the spontaneous LH surge, it may occasionally occur up to 56 h after the LH surge (60). Further, follicular collapse is the most common sign of ovulation (61). Not infrequently (8% in our experience-unpublished data), corpus luteum formation, without follicular collapse (luteinized unruptured follicle syndrome (LUF)) may be noted (62); of importance, serum P_4_ is significantly lower in such LUF cycles when compared to those with follicular rupture despite similar duration of luteal phase (62, 63). Clearly, such sub-optimal mid-luteal serum P_4_ levels, if not monitored, may be associated with worse reproductive outcome.

To conclude, in t-NC cycle, differences in definition of LH surge may result in differences in timing of FET which may have impact on reproductive outcomes. The optimal definition of LH surge associated with the best reproductive outcome should be explored.

#### Comparison of t-NC *Versus* Modified-NC

Three RCTs compare the reproductive outcomes of t-NC with modified-NC. The initial RCT with a limited sample size in patients undergoing cleavage stage FET, reported similar CPR and LBR when comparing t-NC (n=30) to modified-NC (n=25) (64). The second RCT by Fatemi et al. in patients undergoing cleavage stage FET revealed significantly lower OPR with modified-NC (n=63) when compared to t-NC (n=61) (14.3% *vs* 31.1%, p=0.025, respectively) (65). The timing of FET was the same in both groups, i.e., following overnight culture 5 days after the LH surge or hCG administration (65). No LPS was administered in both groups. Of note, if hCG was administered at the time of an impending LH surge (without P_4_ rise), which was encountered in 36.5% (23/63) of the patients, the OPR was disappointingly low (1/23; 4.3%). The study was interrupted prematurely. However, the impact of an impending LH surge on reproductive outcomes in modified-NC is controversial, some reporting a negative impact (66) and others no impact at all (51, 52).

A subsequent retrospective study compared t-NC without LPS (n=501), t-NC with LPS (n=828) and modified-NC with LPS (n=1024) (67). There were several differences between this retrospective study and the RCT by Fatemi et al. (65). First, the timing of FET; since ovulation usually occurs at a later stage after hCG administration (+2 days) compared to ovulation after the spontaneous LH rise (+1 day) (68), warmed blastocyst transfer was scheduled to LH surge+6 days in t-NC and hCG+7 days in the modified-NC (67). Second, both cleavage and blastocyst stage FET cycles were included in the study by Montagut et al. (67). Third, no LPS was administered in the Fatemi et al. ‘s RCT (65). Fourth, patients who were scheduled to have a modified-NC-FET, but subsequently had a spontaneous LH surge were placed in the t-NC or t-NC + LPS groups, depending on whether LPS was added or not in the study by Montagut et al. (67). Despite these differences, the CPR was significantly higher in the t-NC group compared to the modified-NC with LPS (46.9% *vs* 29.7%, respectively, p<0.001), in line with the previous RCT (65).

The most recent RCT, compared t-NC (n=130) to modified-NC (n=130) in a total 260 patients undergoing cleavage stage FET (69). None of the patients received LPS, and following overnight culture, FET was performed at LH surge+5 days in the t-NC group and hCG+6 days in the modified-NC group. In patients assigned to modified-NC, a shift to t-NC was carried out if a spontaneous surge was detected; this was encountered in 28.4% (37/130) of the patients. The CPR, which was the primary outcome measure, was comparable between the modified-NC and t-NC groups (27.2% *vs* 24.4%, respectively; [relative risk (RR)=0.90, 95% CI 0.59-1.37, p = 0.61, respectively]), but with fewer clinic visits for patients undergoing modified-NC (69). The FET timing, shifting to t-NC with impending LH in patients initially assigned to modified-NC and no LPS administration may account for similar reproductive outcome in both groups. However, these results are in line with the previously published retrospective cohort studies (70–73). The results of the ongoing multicenter RCT (Antarctica-2) comparing t-NC and modified-NC will add important information to this field (74).

In a recent network meta-analysis of 26 RCTs, no difference in LBR was reported between t-NC and modified-NC FET (OR=1.21, 95% CI 0.81 – 1.82, low quality evidence) (75).

#### Timing of Embryo Transfer in t-NC and Modified-NC

In a NC, the implantation window ranges between LH+7 to LH+11 (76). A difference in the timing of FET in t-NC versus modified-NC could be considered, as ovulation occurs 36–48 h after hCG administration, but could vary from 24 to 56 h after a spontaneous LH surge (60). The usual practice to perform FET at the blastocyst stage is on LH+6 day in t-NC (and/or ultrasonographic documentation of ovulation +5 day) and hCG+7 day in modified-NC. In a recent retrospective study of 453 single euploid blastocyst transfers using the modified-NC, timing of FET was adjusted based on the presence or absence of a spontaneous LH surge (≥ 20 mIU/mL) (77). With a documented LH surge, FET was scheduled on hCG+6 day (n=205), whereas it was scheduled to hCG+7 day in those cycles without an LH surge (n=248). Similar LBRs were reported for the hCG+6 and hCG+7 groups (61.0% *vs* 60.9%, respectively, p=0.93; adjusted OR=0.98, 95%CI 0.67–1.45) (77).

Moreover, a Scandinavian multi-center RCT is currently ongoing to explore the optimum timing for transferring a single frozen-warmed blastocyst in modified-NC, that is, hCG+6 days or hCG+7 days (78). In this study, each group is further assigned to receive LPS or not. A total of 604 patients is planned to be randomized to four arms.

In conclusion, for warmed blastocyst transfer cycles, based on the available evidence, FET should be timed on LH+6 day in t-NC and hCG+7 day in modified-NC cycles (**Figure 1**).

#### Is LPS Needed in t-NC or Modified-NC?

The need for LPS in t-NC or modified-NC remains equivocal. Optimum P_4_ output from the corpus luteum originating from a mono-follicular spontaneous cycle is crucial for establishing and maintaining an intrauterine pregnancy (79). Until now, there are 2 RCTs evaluating LPS in t-NC, one with exogenous vaginal P administration (80) and the other with hCG administration (81). In the study by Bjuresten et al., 435 women undergoing cleavage stage FET, were randomized to either vaginal P (400 mg vaginal micronized P bid) or no LPS (80). Embryo transfer was performed on the LH surge+3 days and exogenous P_4_ started in the evening of FET. Administration of LPS was associated with a statistically significant increase in LBR (30 *vs* 20%; p = 0.027). In the RCT by Lee et al. (81), 450 women undergoing cleavage stage FET employing the t-NC were randomized to two bolus doses of 1,500 urinary hCG, one on the day of FET and one on FET+6 days, and FET was performed on the third day after the LH surge. The OPR at 10-12 weeks of gestational age, which was the primary outcome measure, was comparable among the two groups (81).

In t-NC, regarding the available retrospective studies some favor LPS (82) whereas others report comparable reproductive outcomes with or without LPS (67, 83, 84).

Since hCG has a long half-life and a sustained luteotropic effect during the early luteal phase up to 7 days following administration (85), LPS might not be needed in modified-NC (86, 87). In line with this two RCTs (87, 88), two retrospective studies report comparable reproductive outcomes with or without LPS in modified-NC (89, 90).

If administered, the timing of LPS administration in a t-NC or modified-NC seems to be important, as too early P administration, may induce embryo-endometrium asynchrony, resulting in impaired reproductive outcomes (67). If LPS is administered, based on the available evidence, it seems that the LPS should not be started earlier than LH surge+3 day (67, 80).

### Mild-Ovarian Stimulation (mild-OS)

Mild OS with an oral agent (CC or letrozole) and/or exogenous gonadotropins may be used to prime the endometrium for FET. For this purpose, mild OS is performed with <150 IU urinary/recombinant follicle stimulating hormone (FSH)/day, letrozole at a dose of 2.5 – 5 mg/day or CC at a dose of 50-100 mg/day, starting on the 2^nd^ or 3^rd^ day of the cycle. The follicular response is monitored by frequent vaginal ultrasonography and/or serum endocrine assessment. Human chorionic gonadotropin is administered when the diameter of the leading follicle is greater than17 mm, endometrial thickness ≥7 mm and serum E_2_ level >150 pg/ml. The timing of the FET is scheduled according to the day of embryo freezing; day-3 embryos are transferred on hCG+5 and day-5/6 embryos are transferred on hCG+7 (91, 92).

The rationale for mild OS in regularly cycling women is to improve subtle defects in folliculogenesis and subsequent luteal phase, resulting in a better endometrial milieu for embryo implantation (91, 92). In addition, mild-OS avoids the reported risks (e.g. thromboembolic events) associated with exogeneous E_2_ and P administration in HRT cycles.

Letrozole is an aromatase inhibitor; it has a half-life of ~2 days compared to ~2 weeks of CC (93). Unlike CC, the hypothalamic-pituitary-ovarian axis is intact during letrozole use. Letrozole has no negative effect on the endometrium (94).

Although the common clinical practice is to employ LPS in mild-OS cycles for FET, mostly extrapolated from non-IVF OS cycles (95), RCTs are clearly warranted to delineate the place for LPS in mild-OS cycles.

### Comparison of Different FET Protocols

#### t-NC/Modified-NC Versus HRT

In a recent Cochrane meta-analysis with pooled analysis of five RCTs, there was a trend, but not reaching statistical analysis, favoring HRT for CPR (OR=0.79, 95% CI 0.62 - 1.01; n=1249; studies=5; I^2^ = 60%; very low-quality evidence). Of the included five RCTs, four [three published in peer review journals (96–98) and one abstract (99)] employed modified-NC and the remaining t-NC (published as an abstract) (100). No difference in LBR was noted between t-NC/modified-NC and HRT (OR=0.97, 95% CI 0.74 - 1.28; n=1285; studies=4; I^2^ = 0%; very low-quality evidence) (5). For comparison of LBR, of the included four RCTs, three employed modified-NC [all three published in peer review journals (96–98)] and the remaining one t-NC (published as an abstract) (100). Of note, the cycle cancellation rate data was available only in one RCT and it was significantly less with modified-NC when compared with HRT protocol (26.7% *vs* 20.4%; p=0.02) (96). The difference in cancellation rates was ascribed mainly to more cancellation due to insufficient endometrial thickness in HRT-FET when compared with modified-NC-FET (OR=13.9, 95% CI 4.4 – 46.7, p<0.01). The Antarctica trial reported similar cost for modified-NC and HRT (96).

Regarding the available retrospective and prospective cohort studies, the majority report similar reproductive outcomes (73, 101–104), whereas better (71, 105, 106) or worse (107, 108) outcomes have also been reported with t-NC/modified-NC when compared to HRT. Importantly, in HRT cycles, the early pregnancy loss rate has been alarmingly high in some reports (73, 109).

In a very recent network meta-analysis of 26 RCTs, regarding LBR, HRT ranked as the worst (albeit not reaching statistical significance), when compared with t-NC (OR= 0.85, 95% CI 0.48 – 1.49) and modified-NC (OR= 0.79, 95% CI 0.56 – 1.11) (75). In the same study, the authors also did a pairwise meta-analysis of 113 observational studies; HRT was associated with a significantly lower LBR when compared with t-NC (OR= 0.81, 95% CI 0.70 – 0.93) or modified-NC (OR= 0.85, 95% CI 0.77 – 0.93) (75).

In conclusion, the currently available low-quality evidence points toward the NC (t-NC/modified-NC) being superior to HRT.

#### Mild-OS Versus HRT

In a recent Cochrane meta-analysis, when stimulation with gonadotropins, letrozole or CC was pooled, the CPR was significantly higher with mild-OS when compared to HRT cycles (OR=1.63, 95% CI 1.12 – 2.38; n=656; five RCTs; I^2^ = 11%; low-quality evidence) (5). In the subgroup analysis, the CPR was significantly higher with letrozole when compared to HRT (OR=1.94, 95% CI 1.24 – 3.04; n=365; 3 RCTs; I^2^ = 15%; low-quality evidence). The LBR was comparable with letrozole and HRT in the only available RCT including a limited sample size (OR=1.26, 95% CI 0.49 - 3.26; n=100; one study; very low-quality evidence) (5, 110). The CPR was comparable with CC (111) or gonadotropins (112) when compared with HRT with no data on LBR.

In a recent network meta-analysis of 26 RCTs comparing different FET protocols, when compared with HRT, significantly higher LBRs were reported with mild-OS using gonadotropin (OR=1.77, 95%CI 1.06 – 2.98; very-low quality of evidence) or mild-OS using letrozole (OR=1.67, 95%CI 1.22 – 2.28; low quality of evidence) (75). However, no significant difference in LBR was noted between t-NC, modified-NC and mild-OS protocols (75).

The endometrial thickness was significantly reduced with CC (Mean Difference= - 1.04, 95% CI -1.59 to -0.49; n=92; one study; low-quality evidence) compared to HRT (5). The endometrial thickness, on the day of starting exogenous P as well as on the day of FET, was significantly higher in the letrozole group when compared to HRT in a recent retrospective study of 2,664 patients with PCOS undergoing FET (113). However, two other small-scaled studies reported no significant difference in endometrial thickness comparing mild-OS with letrozole to HRT cycles (110, 114).

In conclusion, although HRT and NC (t-NC/modified-NC) are the most commonly used protocols, recent emerging evidence suggests that mild-OS may be a viable option for FET.

## Individualized FET Approach

### Endocrine Monitoring

Personalized medicine is crucial to maximize efficacy, safety and minimize treatment burden, and priming of the endometrium for FET, is obviously not an exception. An optimal exposure of the endometrium to P in terms of timing and concentration, is essential to maximize the reproductive outcome of the HRT cycle. In theory, the use of endometrial P_4_ measurements would be ideal, however, this is not feasible in clinical practice, and as such the serum P_4_ level, which is impacted by the route of administration, is still the best proxy for the endometrial progesterone level (115). Until recently, “im-personalized”, standard LPS without luteal serum P_4_ monitoring was the” standard of care” and the typical practice for HRT cycles in IVF programs. However, recently available evidence clearly indicates that, in HRT cycles, there might be marked inter-personal variation in serum P_4_ levels with an impact on reproductive outcomes, despite the use of the same dose and route of P administration (36, 116–128). Although these studies were heterogenous in population, regarding the type, dose, and mode of P administration, of the embryo stage at transfer, and the day of serum P_4_ measurement, better reproductive outcomes were reported when the serum P_4_ level was above a certain threshold, ranging from 8.75 to 32.50 ng/ml (36, 116–120, 122–124, 126, 127) (**Table 2**).

**Table 2:** Overview of studies on serum progesterone (P) monitoring in HRT cycles.

Reference	n	Route of P	Dose of P	Optimal P level/P test day	Site/No. of embryo transfer/embryos	Outcome, % (high *vs* low P group)
Brady et al., 2014 (118)	229	im	50-100 mg x 1	>20 ng/ml (64 nmol/l)/5^th^ P day	Single center/1 to >3 fresh donor Day 3 embryos	LBR (65 *vs* 51)
Kofinas et al., 2015^a^ (121)	213	im	50-75 mg x 1	<20 ng/ml (64 nmol/l)/6^th^ P day	Single center/SET/vitrification, euploid blastocyst, autologous	LBR (65 *vs* 49)
Yovich et al., 2015^a^ (124)	529	Vaginal	400 mg x3 (in-house produced pessaries)	22.1 – 31.2 ng/ml (70 – 99 nmol/l)/6^th^ P day	Single center/SET/vitrification, blastocyst, autologous + donor	LBR (50 *vs* 41)
Labarta et al., 2017 (122)	211	Vaginal	400 mg x 2 (micronized P)	>11 ng/ml (>35 nmol/l)/6^th^ P day	Single center/SET or DET/vitrification, blastocyst, donor	OPR (53 *vs* 43)
Basnayake et al., 2018 (117)	1580	Vaginal	Various	>15.8 ng/ml (>50 nmol/l)/6^th^ P day	Multicenter/SET/cleavage or blastocyst, slow freeze or vitrification, donor + autologous	LBR (27 *vs* 11)
Alsbjerg et al., 2018 (116)	244	Vaginal	90 mg x 3 (bioadhesive P gel)	≥11 ng/ml (≥35 nmol/l)/9^th^ – 11^th^ P day	Single center/SET or DET/blastocyst, vitrification, autologous	OPR (51 *vs* 38)
Gaggiotti-Marre et al., 2019 (120)	244	Vaginal	200 mg x 2 (micronized P)	>10.64 ng/ml/4^th^ P day	Single center/preferably SET/vitrification, euploid blastocyst, autologous	LBR (62 *vs* 48)
Cédrin-Durnerin et al., 2019 (119)	227	Vaginal	200 mg x 2 (micronized P)	≥10 ng/ml/2^nd^, 3^rd^ or 5^th^ P Day	Single center/SET or DET/Day 2 or 3 or blastocyst, autologous	LBR (31 *vs* 17)
Boynukalin et al., 2019 (127)	168	im	100 mg/daily	≥20.6 ng/ml/6^th^ P day	Single center/Single euploid blastocyst transfer/Day5 biopsied blastocysts, vitrification	OPR (70 *vs* 42)
Polat et al., 2020 (36)	143	Vaginal	90 mg x 2 (bioadhesive P gel)	>8.75 ng/ml/5^th^ P day	Single center/SET or DET/vitrification, blastocyst	OPR (47 *vs* 29)
Alyasin et al., 202l^a^ (126)	258	Vaginal and im	400 mg x 3 (micronized P) and 50mg/daily/im	<32.5 ng/ml/5^th^ P day	Single center/SET or DET/vitrification, blastocyst	LBR (42 *vs* 23)

a study reporting detrimental effect of high progesterone level on reproductive outcome.

P, Progesterone; SET, Single embryo transfer; DET, Double embryo transfer; LBR, Live Birth Rate; OPR, Ongoing Pregnancy Rate; im, intramuscular.

It remains to be determined whether a ceiling level of serum P_4_ in HRT cycles exists above which reproductive outcomes are impaired. A ceiling effect was reported in three HRT studies (121, 124, 126), whereas no significant effect was reported in the remaining 8 (36, 116–120, 122, 127).

In the Kofinas et al.’s study, including 213 patients undergoing euploid blastocyst transfer and HRT with im P, a ceiling effect was reported with serum P_4_ levels over 20 ng/ml on the sixth day of P start (121). In the Yovich et al. study, using a homemade pessary, an optimal serum P_4_ window of 22.01–31.1 ng/ml on the sixth day of P start was reported, whereas patients with serum P_4_ levels exceeding 31.1 ng/ml had poorer implantation rates and LBRs (124) (n=539). In a very recent study, a ceiling effect was noted for CPR and LBR with serum P_4_ levels ≥ 32.5 ng/ml (126). In our recent study of 475 FET cycles, no ceiling effect was noted on OPR in either the vaginal gel only or vaginal gel and im P supplemented groups (36).

Since a corpus luteum is absent in HRT-FET, the marked inter-personal differences in serum P_4_ might be caused by altered pharmacokinetics (uptake/distribution and metabolism) of the exogenous P (129), affected by body mass index (BMI) (118, 125), female age (125, 130), as well other intrinsic patient factors (125).

Unlike serum P_4_, no association has been noted between late-proliferative phase serum E_2_ levels and LBR in HRT cycles (131).

### Rescue Protocol

A natural question to ask regarding patients with low serum P_4_ levels, is whether the cycle can be “rescued” with additional exogenous P, using the same or a different route of administration. In an oocyte donation study by Brady et al. (n=229 cycles), im P was administered at a dose of 50–100 mg per day, commencing in the evening and 4 days before cleavage stage embryo transfer (118). Serum P_4_ was measured on the day of embryo transfer and if P was <20 ng/mL, the dose of im P was increased by 50–100%. The threshold of 20 ng/ml represented the cut-off value between the first and second tertiles of serum P_4_ on the day of embryo transfer. Additional P dosing was necessary in a total of 32.8% of the cycles (75 of 229). However, despite supplementation, the LBR was still lower compared to patients with serum P_4_ >20 ng/ml (52.0% *vs* 64.9%, p=0.04) (118).

In the study by Cedrin-Durnerin et al., a total of 195 patients undergoing 227 FET cycles in an HRT protocol were included (119). Micronized vaginal P, 200 mg three times daily was used for LPS. Embryo transfer was performed after the morning administration of P, on day 2 of P administration for day 2 embryos, on day 3 for day 3 embryos and on day 5 for blastocysts. Serum P_4_ was measured immediately before embryo transfer in all patients. If the serum P_4_ level was less than 10 ng/ml (n=85 patients), patients were contacted in the afternoon to increase the P dose to 400 mg three times daily and a new blood sample was performed two days later to check the serum P_4_ level. The repeat serum P level two days later, available in 80 patients, showed an increase in serum P_4_ level >10 ng/ml in 55 (69%) patients, only. However, the LBR in patients who had increased serum P_4_ levels of >10 ng/ml after increased dosing were not statistically different from those in whom serum P_4_ levels remained <10 ng/ml (20% and 12%, respectively). Moreover, the LBR of this group of patients was overall lower than in patients with serum P_4_ >10 ng/ml group who did not require supplementation (119).

Notwithstanding the above findings, these two studies have several limitations (118, 119). First, both studies were retrospective with inherent selection bias. Second, LPS “rescue” was not the main research question in the studies (118, 119), and without performing multivariate logistic regression analysis, it is not possible to accept or refute the role of rescue in these cycles. Third, even with the rescue protocol, the lowest serum P_4_ level below which cycle cancellation was undertaken was not reported in the available two studies (118, 119).

In a very recent prospective cohort study, the impact of rescue on the reproductive outcome was evaluated in 453 women undergoing 574 euploid blastocyst transfer in an HRT protocol (128). For serum P_4_, a cut-off point of 10.6 ng/ml, one day prior to embryo transfer, was taken as the threshold below which rescue was performed by administering a supplementary dose of 25 mg sc P. Cycles with serum P_4_ >10.6 ng/ml served as the controls (n=348). Of the 574 cycles included, rescue was necessary in a total of 226 cycles (39.4%). Embryo transfer was performed if serum P_4_ levels exceeded 10.6 ng/ml on the day of FET (one day after rescue) which was the case in 98.2% of cycles. The LBRs of the control and rescue groups were comparable (49.1% *vs* 52.3%: respectively, RD −3.2%, 95% CI − 12; 5.7). Moreover, no significant difference was noted in the miscarriage rate (12.4% *vs* 9.2%, respectively; RD=3.2%, 95% CI − 4.3; 10.7) (128).

We recently reported rectifying OPRs with a rescue protocol in patients undergoing HRT FET and low serum P_4_ concentrations one day prior to FET (132) Vaginal bioadhesive gel 90 mg bid was used for LPS. Employing a case–control study design, 40 patients with low serum P_4_ concentrations (<8.75 ng/ml) on the 5^th^ day of P supplementation underwent rescue with a daily bolus of 25 mg s.c. P, starting on the afternoon of the 5^th^ day of P administration. For every patient who underwent P rescue, three patients matched by age, body mass index, number of previous attempts and number of blastocysts transferred, with serum P_4_ concentration >8.75 ng/ml on the 5^th^ day of P administration served as controls (n = 120). Following rescue, the mean serum P_4_ concentration on the day of vitrified–warmed embryo transfer (6^th^ day of P administration) was rectified in all patients and was 33.43 ± 10.83 ng/ml (range 14.61–82.64 ng/ml). The OPR of the rescue and control groups were comparable (50% *vs*. 48.3%, p=0. 853, respectively) (132).

It is reassuring that the study by Alvarez et al. (128) and ours (132), the first two studies of its kind, are concordant and support rectifying reproductive outcome by a rescue protocol of a daily bolus of 25 mg s.c. P, starting on the afternoon of the 5^th^ day of P administration. However, some differences between two studies still exist: i) type of vaginal P used: micronized vaginal P at a dose of 200 mg/8 hours in the study by Alvarez et al. and vaginal bioadhesive gel 90 mg/12 hours in our study; ii) no fixed timing for P (vaginal/sc P) administration and blood withdrawal in the study by Alvarez et al. in contrast to 4-5 hours after morning administration of vaginal gel in our study; iii) FET was scheduled on P start+6 day in both studies; however, in the study by Alvarez et al., the nightly administration of P (200 mg) was the dose for the first day of P administration. Hence, the patients apparently did not receive the full dose of 200 mg/8 hr on the first day of P administration; iv) minimum P_4_ level below which cycle cancellation was undertaken: it was not reported in the study by Alvarez et al. but was set as 4 ng/ml (albeit arbitrary) on the 5^th^ day of P start, in our study; v) the threshold of 10.6 ng/ml in their study is the interception of second and third quartiles (120), whereas, our threshold of 8.75 ng/ml was the 10% of our previous study (36).

Further RCTs are warranted, exploring different types and routes of P to firmly establish the success of the rescue protocol. Finally, the lowest serum P_4_ level suitable for rescue should also be defined.

Using a threshold of 10 ng/ml during the mid-luteal phase of natural cycles (133), a luteal phase defect was reported in 8% of normo-ovulatory subfertile women (134). To our knowledge, until now, only one study reported the impact of serum P_4_ one day prior to warmed blastocyst transfer in t-NC cycles (135). In this study, a total of 294 t-NC FET cycles, were included; of those 294 cycles a total of 86 (29.2%) were PGT-A cycles. No LPS was administered. Patients were divided into two groups according to serum P_4_ levels, below or above 10 ng/ml on the day before FET. Of note, 37% patients had a serum P_4_ level <10 ng/ml. Patients in the higher P_4_ group (>10 ng/ml) had higher CPR (48.6% *vs* 33.0%: RD=15.6%, 95% CI 4-27) and LBR (41.1% *vs* 25.7%: risk difference (RD)= 15.4%, 95% CI 5–26) than those with P_4_ levels <10 ng/ml. Of note, patients with serum P_4_ levels <10 ng/ml on the day before FET had a significantly higher weight and BMI compared to patients with serum P_4_ levels >10 ng/ml, and obviously the pulsatile secretion of P_4_ is a limitation of one single measurement. Whether cycle cancellation or rescue with P should be undertaken in cycles with low serum P_4_ needs to be further evaluated.

### Assessment of Window of Implantation (WOI)

Despite the transfer of good quality blastocysts, implantation failure due to an endometrial factor still remains a challenge for both physicians and embryologists. The endometrium is receptive during the window of implantation (WOI) regulated by an incompletely understood endocrine, paracrine, and autocrine factors (136). During the natural cycle, the WOI is limited to days 8-10 after ovulation during which the blastocyst may implant (137). However, there seems to be marked inter-personal differences in the timing of the WOI that cannot be elucidated by ultrasonographic, hormonal or histologic assessments (138). Recently, a commercial endometrial receptivity array (ERA) test was brought to the market as a new tool to define the WOI, particularly in patients with recurrent implantation failure, permitting personalized timing of the embryo transfer (pET). Of interest, 12% of good prognosis patients (normal ovarian reserve and at least 6 mature oocytes retrieved) have been reported to have an altered WOI (139). The ERA test evaluates the 238 transcriptomic signature genes in a timely sampled endometrial tissue by a machine learning algorithm to identify the endometrial sample’s receptivity status on the day of biopsy (140).

In this aspect, a recently published multicenter, open-label RCT explored pET policy in 458 good prognosis women (female age ≤body-mass index of 18.5-30.0 kg/m^2^, antral follicle count ≥8 and FSH <8 IU/L) undergoing blastocyst transfer (141). Patients were randomized to pET guided by ERA (n=148), FET (n=154) or fresh embryo transfer (n=156) in 16 clinics. Although reproductive outcomes by intention-to-treat analysis were comparable, the cumulative LBRs after 12 months were 71.2% in pET versus 55.4% in FET (p= 0.04), and 48.9% in fresh embryo transfer (p=0.003) groups. Clinical pregnancy rates after the first embryo transfer were higher with pET compared with FET (72.5% *vs* 54.3%, respectively, p = 0.01) and fresh embryo transfer (72.5% *vs* 58.5%, respectively; p = 0.05) groups.

Until now, however, the place of ERA testing in FET cycles is controversial as three recent studies refuted any potential benefit of ERA testing (142–144) whereas one study reported more favorable outcomes, however, not reaching statistical significance (145). In the study by Riestenberg et al, a total of 228 single euploid FET HRT cycles were either guided by ERA testing (pET) (n=147) or by standard timing without ERA (n=81) (144). The LBR did not differ between patients who underwent FET with ERA/pET, 45/81 (56.6%) or standard timing 83/147 (56.5%) (144).

In conclusion, the available data on ERA testing for personalized timing of embryo transfer are conflicting and warrant further studies.

### Endometrial Thickness and Thin Endometrium

An endometrial thickness of 7 mm is generally considered to be the cut-off for a “thin” endometrium, below which many physicians would cancel an embryo transfer (146, 147). In the recent Canadian Database of 12,433 FET cycles, thin endometrium has been reported in ~3% of the cycles (147); both CPR and LBR decreased significantly when the endometrial thickness was below 7 mm on the day of P start or documentation of LH surge. The LBRs with endometrial thickness of 5.0-5.9 mm (71 cycles), 6.0-6.9 mm (290 cycles), 7.0-7.9 mm (848 cycles) and mm (9860 cycles) were 16.9%, 31.7%, 33.3% and 40.6%, respectively. No live birth occurred in nine patients with endometrial thickness <5mm (147).

In contrast to the Canadian database study, a recent small-scaled retrospective study of 287 HRT cycles reported no linear association between endometrial thickness, LBR and miscarriage rate (148). Area under the curve values for endometrial thickness to predict LBR was 0.47 (148).

Thin endometrium remains to be a challenge to overcome. Several strategies have been proposed to manage thin endometrium in FET cycles, including modification of E_2_ use (149–151), in-utero administration of granulocyte colony stimulating factor (152), in-utero administration of platelet-rich plasma (153), vaginal sildenafil (154), oral pentoxyfiline, oral tocopherol (155), oral aspirin (156) or pelvic floor neuromuscular electrical stimulation (157). However, none of these strategies have been unequivocally proven to be of benefit in patients with thin endometrium (158).

Three modifications on the administration of E_2_ have been investigated in patients undergoing HRT FET with thin endometrium (158): i) increasing the dose and duration of E_2_ administration. In the only available prospective case-control study, of the 36 patients with a history of refractory thin endometrium and planned to undergo fresh embryo transfer, 23 had fresh embryo transfer despite thin endometrium and the remaining 13 patients had HRT FET with an extended E_2_ therapy (149). A significant increase in endometrial thickness (6.7 ± 0.9 mm in fresh transfer cycles *vs*. 8.6 ± 0.7 mm in HRT FET cycles; p=0.031) and CPR (4.3% in fresh transfer cycles *vs*. 38.5% in HRT FET cycles, p = 0.016) was noted following an extended E_2_ therapy lasting 14 to 82 days (mean=30 days) (149); ii) changing the route of administration of E_2_. While oral route is widely prescribed due to its simple and well-tolerated use, parenteral routes may increase serum E_2_ concentration through bypassing the first-pass hepatic metabolism (159). Moreover, vaginal administration of E_2_ is associated with first-pass uterus effect (160). Thus, in theory, vaginal administration, either alone or combined with oral administration, may be a viable option in patients with thin endometrium. A retrospective study of 247 patients undergoing HRT-FET with thin endometrium compared extended oral E_2_ administration-only (n=69) with extended oral & vaginal E_2_ administration (1-2 mg/day) (n=178) (150). Although extended oral & vaginal E_2_ administration was associated with a significantly thicker endometrium, CPRs of the two groups were comparable (150). In contrast, an increase in endometrial thickness by supplementing with vaginal E_2_ (4 mg/day) was refuted by a prospective trial (161). Subcutaneous E_2_ pellets may be used for thin endometrium as was reported by a small scaled oocyte donation study (162); iii) modification of the type of E_2_. A RCT compared vaginal synthetic estrogen (n=30) with vaginal natural estrogen (n=30) in patients with refractory thin endometrium. Endometrial thickness in the synthetic E_2_ group was significantly higher when compared with the natural E_2_ group (5.93 ± 0.38 *vs*. 6.74 ± 0.32; respectively, p<0.001) (151); of note, reproductive outcomes have not been reported. Clearly, more well-designed trials are warranted to delineate the role of modification of E_2_ administration in patients with refractory thin endometrium.

The impact of thin endometrium on reproductive outcome may go beyond the chance of conception since thin endometrium may be associated with adverse obstetric and neonatal outcomes (163, 164). In a very recent study of 13,383 FET cycles (HRT and NC), an increasing risk of preterm delivery and low birth weight has been reported with thin endometrium (<8 mm) (164).

Recently, in HRT cycles, endometrial compaction, as defined by a decrease in endometrial thickness at the time of warmed blastocyst transfer when compared to the day of P start, was reported to be a favorable factor for OPR when compared to cycles with no change or increase in endometrial thickness (165, 166). Of note, endometrial compaction was seen in 69% (187/271) HRT-FET cycles (165). The authors suggested that failure of endometrial compaction may be caused by an inadequate response to P or to P resistance (167) which may be overcome, in theory, by increasing the dose or duration of P during the luteal phase and/or decreasing exogenous or endogenous estrogen. The authors further speculated that endometrial compaction may be an important biomarker in fresh, t-NC and modified-NC FET. However, several recent large-scale studies refuted the importance of endometrial compaction in HRT (168–170), modified-NC (168, 169, 171) and even fresh embryo transfer cycles (172).

In conclusion, endometrial thickness below 7 mm (5-7 mm) is a weak predictor of reproductive outcome in FET cycles. However, although rare, very thin endometrium (<5mm) is associated with futile outcome. The limited available evidence does not support any particular treatment modality to be of benefit in patients with thin endometrium. Endometrial compaction is not a predictor of reproductive outcome in FET cycles.

### FET Cycles in Patients With Endometriosis

Biological differences exist in eutopic endometrium of patients with and without endometriosis (173–175) which may contribute to altered endometrial receptivity (176, 177). In a matched cohort study by Bourdon et al. (178), including a total of 270 endometriosis patients undergoing either fresh embryo or frozen embryo transfer, the authors reported a doubling of the cumulative OPR in patients undergoing FET as compared to fresh transfer (34.8% *vs* 17.8%), clearly showing a benefit of FET in this subgroup of patients (178, 179). To our knowledge, there is no RCT comparing different FET protocols in patients affected with endometriosis. An extended gonadotropin suppression therapy of 2 months before the FET-HRT cycle may benefit such patients (180). However, sufficiently powered RCTs are warranted to confirm such a policy.

### FET Cycles in Patients With Polycystic Ovary Syndrome (PCOS)

Mild-OS and HRT are the two most commonly applied FET protocols in patients with PCOS; however, there is no RCT comparing the reproductive outcomes of these two protocols.

In a recent meta-analysis of four retrospective studies, mild-OS (letrozole or low dose gonadotropins) was compared with HRT in PCOS patients (181). No significant difference was noted in OPR, 52.1% with mild-OS and 47.3% with HRT (4 studies, n= 2,933; OR= 1.12, 95% CI, 0.92-1.35; I^2 =^ 59%). Similarly, LBR, CPR, implantation and miscarriage rates were comparable. However, in a subgroup analysis, a lower miscarriage rate was noted in letrozole-stimulated cycles than HRT cycles (3 studies; OR= 0.53; 95% CI 0.40–0.70; I^2 =^ 0%) (181). These intriguing findings need to be confirmed by future RCTs.

## Maternal and Obstetric Outcomes After Different FET Protocols

Hypertensive disorders in pregnancy are significantly increased following an HRT cycle compared with NC or mild OS-FET cycle (182–185). The increased risk may relate to the absence of a corpus luteum leading to i) decreased serum relaxin and vascular endothelial growth factor levels ii) lower reactive hyperemia index, iii) lower angiogenic and nonangiogenic circulatory endothelial progenitor cells iv) a lack of drop in mean arterial pressure during pregnancy (186–189). Moreover, the risk of postpartum hemorrhage, and cesarean section is significantly increased after HRT compared with t-NC or modified-NC (182, 183, 185). Macrosomia and large for gestational age (LGA) are increased in FET cycles when compared with fresh embryo transfer cycles (190) and spontaneous pregnancies (191). The type of FET protocol may be a determining factor for the development of macrosomia/LGA. Thus, in a large retrospective cohort study of 9267 FET cycles, resulting in singleton live birth, the mean birthweight of different FET protocols was compared, including modified-NC (n=2224), mild-OS (n=4299) and HRT (n=2744). A significantly higher mean birthweight was reported in the HRT group when compared with mild-OS. Singleton newborns conceived after FET using HRT were more likely to be LGA than those born after modified-NC or mild-OS (19.92% vs 16.94% and 19.29% vs 16.12%, respectively). The mild-OS group had lower adjusted odds of being macrosomia than the modified-NC group. No significant difference was noted for birthweight, and risk of macrosomia/LGA between modified-NC and mild-OS (192). Furthermore, a significantly higher risk for birth weight >4,500 g was observed by other large-scale studies with the HRT protocol than with the modified-NC regimen (183, 185). A large retrospective Nordic register-based cohort study reported that, when compared with natural conceptions, the mean birth weight of FET pregnancies become significantly higher starting from gestational week 33 among boys and from gestational week 34 among girls (193).

Regarding obstetric outcomes, the risk of preterm delivery, very preterm delivery and premature rupture of the membrane increase with the HRT protocol as compared to modified-NC (185). In contrast, there appears to be no statistically significant difference in the rate of small for gestational age, placenta previa, or congenital abnormality among modified-NC, mild-OS and HRT protocols (185). Similar neonatal outcome has been reported between HRT and modified-NC cycles (182).

With all given increased maternal and obstetric risk factors, some authors suggested a “back to nature” attitude, advocating for more t-NC and modified-NC in future (194).

## Conclusions and Future Perspectives

Despite the worldwide increase in FET for various indications, the search for the best protocol to prepare the endometrium continues. Although HRT and NC (t-NC/modified-NC) are the most commonly used protocols, recent emerging evidence also suggests that mild-OS may be a viable option. Well-designed, powerful RCTs comparing different protocols to prime the endometrium for FET are urgently required; such trials should focus not only on LBR, but also on maternal, obstetrical and neonatal outcomes. Currently, low quality evidence points toward the NC (t-NC/modified-NC) being superior to HRT. Furthermore, in HRT cycles, caution is warranted since the early pregnancy loss rate seems to be alarmingly high in some reports and recent evidence indicates an increased risk of hypertensive disorders in pregnancy in cycles without a corpus luteum. Regarding warmed blastocyst transfer and timing, the evidence suggests 6^th^ day of P start, LH surge+6 day and hCG+**7** day in HRT, t-NC and modified-NC, respectively. Time corrections, due to inter-personal differences in the WOI or day of vitrification (day 5 or 6), should be explored further. In the era of personalized medicine, the “one size fits all” strategy does not match the FET cycle. Although not ideal and affected by the route of administration, serum P_4_ level is a good proxy of endometrial P. A significant inter-personal variation in circulating P in HRT and NC cycles exists and these variations have a major impact on the reproductive outcome. Finally, the place of P rescue protocols in patients with low serum P_4_ levels one day prior to warmed blastocyst transfer in HRT and NC- FET is likely to be intensively explored in near future.

## Author Contributions

SM and HY was involved in the review design, execution, manuscript drafting, and critical discussion of the manuscript. PH, SE, and EP was involved in the execution, manuscript drafting, and critical discussion of the manuscript. MP, IY, and GB was involved in manuscript drafting, and critical discussion of the manuscript. All authors contributed to the article and approved the submitted version.

## Conflict of Interest

HY, EP, and GB declare receipt of honorarium for lectures from Merck and research grants from Merck and Ferring. SE declares receipt of unrestricted research grants from Merck and lecture fees from Merck and Med.E.A. PH has received unrestricted research grants from MSD and Merck, as well as honoraria for lectures from MSD, Merck, Gedeon–Richter, Theramex, and IBSA.

The remaining authors declare that the research was conducted in the absence of any commercial or financial relationships that could be construed as a potential conflict of interest.
